# Microsatellites reveal high polymorphism and high potential for use in anti-malarial efficacy studies in areas with different transmission intensities in mainland Tanzania

**DOI:** 10.1186/s12936-024-04901-6

**Published:** 2024-03-15

**Authors:** Deus S. Ishengoma, Celine I. Mandara, Rashid A. Madebe, Marian Warsame, Billy Ngasala, Abdunoor M. Kabanywanyi, Muhidin K. Mahende, Erasmus Kamugisha, Reginald A. Kavishe, Florida Muro, Renata Mandike, Sigsbert Mkude, Frank Chacky, Ritha Njau, Troy Martin, Ally Mohamed, Jeffrey A. Bailey, Abebe A. Fola

**Affiliations:** 1 National Institute for Medical Research, Dar es Salaam, Tanzania; 2 Faculty of Pharmaceutical Sciences, Monash University, Melbourne, Australia; 3 Harvard T.H. Chan School of Public Health, Harvard University, Boston, MA USA; 4 Gothenburg University, Gothenburg, Sweden; 5 Department of Parasitology, School of Public Health, Muhimbili University of Health and Allied Sciences, Dar es Salaam, Tanzania; 6 Department of Women’s and Children’s Health, International Maternal and Child Health (IMCH), Uppsala University, Uppsala, Sweden; 7 Ifakara Health Institute, Dar es Salaam, Tanzania; 8 Bugando Medical Centre, Catholic University of Health and Allied Sciences, Mwanza, Tanzania; 9 Kilimanjaro Christian Medical Centre, Kilimanjaro Christian Medical University College, Moshi, Tanzania; 10 National Malaria Control Programme, Ministry of Health, Dodoma, Tanzania; 11 Malariologist and Public Health Specialist, Dar es Salaam, Tanzania; 12 HIV Vaccine Trials Network, Fred Hutch Cancer Research Centre, Seattle, WA USA; 13 Department of Pathology and Laboratory Medicine, Warren Alpert Medical School, Brown University, Providence, RI USA

**Keywords:** *Plasmodium falciparum*, Malaria, Therapeutic efficacy studies, Microsatellites, Tanzania

## Abstract

**Background:**

Tanzania is currently implementing therapeutic efficacy studies (TES) in areas of varying malaria transmission intensities as per the World Health Organization (WHO) recommendations. In TES, distinguishing reinfection from recrudescence is critical for the determination of anti-malarial efficacy. Recently, the WHO recommended genotyping polymorphic coding genes, merozoite surface proteins 1 and 2 *(msp1 and msp2)*, and replacing the glutamate-rich protein (*glurp*) gene with one of the highly polymorphic microsatellites in *Plasmodium falciparum* to adjust the efficacy of antimalarials in TES. This study assessed the polymorphisms of six neutral microsatellite markers and their potential use in TES, which is routinely performed in Tanzania.

**Methods:**

*Plasmodium falciparum* samples were obtained from four TES sentinel sites, Kibaha (Pwani), Mkuzi (Tanga), Mlimba (Morogoro) and Ujiji (Kigoma), between April and September 2016. Parasite genomic DNA was extracted from dried blood spots on filter papers using commercial kits. Genotyping was done using six microsatellites (Poly-α, PfPK2, TA1, C3M69, C2M34 and M2490) by capillary method, and the data were analysed to determine the extent of their polymorphisms and genetic diversity at the four sites.

**Results:**

Overall, 83 (88.3%) of the 94 samples were successfully genotyped (with positive results for ≥ 50.0% of the markers), and > 50.0% of the samples (range = 47.6–59.1%) were polyclonal, with a mean multiplicity of infection (MOI) ranging from 1.68 to 1.88 among the four sites. There was high genetic diversity but limited variability among the four sites based on mean allelic richness (R_S_ = 7.48, range = 7.27–8.03, for an adjusted minimum sample size of 18 per site) and mean expected heterozygosity (*H*_*e*_ = 0.83, range = 0.80–0.85). Cluster analysis of haplotypes using STRUCTURE, principal component analysis, and pairwise genetic differentiation (*F*_*ST*_) did not reveal population structure or clustering of parasites according to geographic origin. Of the six markers, Poly-α was the most polymorphic, followed by C2M34, TA1 and C3M69, while M2490 was the least polymorphic.

**Conclusion:**

Microsatellite genotyping revealed high polyclonality and genetic diversity but no significant population structure. Poly-α, C2M34, TA1 and C3M69 were the most polymorphic markers, and Poly-α alone or with any of the other three markers could be adopted for use in TES in Tanzania.

**Supplementary Information:**

The online version contains supplementary material available at 10.1186/s12936-024-04901-6.

## Background

Malaria case management is one of the main interventions for malaria control, and together with vector control tools, it has significantly contributed to the reduction in morbidity and mortality that was reported between 2000 and 2015 [1]. However, this strategy has been compromised by antimalaria drug resistance, which led to the withdrawal of chloroquine and sulfadoxine–pyrimethamine (SP) and the replacement of these drugs with artemisinin-based combination therapy (ACT) [2]. In 2006, Tanzania introduced ACT with artemether–lumefantrine (AL) for the treatment of uncomplicated malaria, and the drug was officially rolled out in January 2007 [3]. AL, which is a fixed-dose combination of artemether and lumefantrine, has been effectively used for the past 16 years for the treatment of uncomplicated falciparum malaria [4], and studies undertaken in Tanzania have shown that it has maintained high and optimal efficacy and safety with high cure rates and minimal safety concerns [5–9]. Previous reports have shown that artemisinin partial resistance (ART-R) emerged in the Mekong Sub-region of South-east Asia following the deployment of ACT and was associated with delayed parasite clearance [10, 11], extended survival at the ring stage [12, 13] and mutations in the *kelch13* (*k13*) gene [14–16].

Until 2018, mutations associated with ART-R had not been reported in Africa [4], and ACT retained high cure rates for the treatment of uncomplicated *Plasmodium falciparum* malaria [4]. However, recent studies showed confirmed ART-R in Rwanda with mutations at codon R561H (> 5%) of the *k13* gene and day 3 positivity rates (> 10%), but AL still had sufficient cure rates (> 90%) [17, 18]. Similarly, ART-R has been reported in Uganda with mutations in the *k13* gene at codons A675V and C469Y [19], in Tanzania with R561H mutations [20] and Eritrea with mutations at codon R622I [21]. For lumefantrine, studies conducted in Tanzania [9] and elsewhere have reported an increase in polymorphisms in the *multidrug resistance 1* gene (*mdr1*), which is associated with reduced susceptibility to lumefantrine [22]. The impacts of the polymorphism (N86/184F/D1246, NFD) in the *mdr1* gene on AL performance are not clear; thus, sustained surveillance is needed to monitor the performance of this important ACT and allow early detection of any emergence of resistance before its efficacy is compromised.

In Tanzania, the National Malaria Control Programme (NMCP) and its partners have been collaboratively implementing therapeutic efficacy studies (TES) since 1997 [23, 24]. These TES are based on the World Health Organization (WHO) standard protocol [25] and aim at monitoring the efficacy and safety of anti-malarials used for the treatment of uncomplicated malaria in children aged 6 months to 10 years. For Tanzania, studies have focused on the first-line anti-malarial (AL) and alternative artemisinin-based combinations. The current alternative ACT covered in TES include artesunate–amodiaquine (ASAQ), which is the first-line drug used in Zanzibar [26], and dihydroartemisinin–piperaquine, which was included in the National Guidelines for Diagnosis and Treatment of Malaria from 2014 [27].

According to the WHO protocol [25], TES has two components: field data and sample collection and laboratory analyses. The laboratory analyses aim at distinguishing recrudescent from new infections in patients with recurrent infections and generating data on molecular markers of the genes associated with resistance or reduced sensitivity/susceptibility of the parasites to the drugs. To distinguish recrudescent from new infections, the old WHO protocol, which was developed in 2007, recommends genotyping three polymorphic genes including merozoite surface proteins 1 and 2 *(msp1 and msp2)* and glutamate-rich protein (*glurp*) [28]. Recently, the WHO recommended a new protocol in which both *msp1* and *msp2* are genotyped together with one or two highly polymorphic microsatellite markers which replace the *glurp* gene because it is not polymorphic enough and has led to underestimation of drug efficacy [29].

Several microsatellite markers have been utilized in studies of malaria parasites, but they differ in their level of polymorphism and informativeness, and their polymorphisms vary among different parasite populations [30]. Of the different microsatellites, the WHO recommends using poly-α and any of the other two markers, TA1 and PfK2. However, these markers have not been optimized in different countries, including Tanzania, to determine whether they are indeed sufficiently polymorphic and sensitive and can reliably be used for genotyping within the TES. This study was therefore undertaken to assess the polymorphisms and genetic diversity of six microsatellite markers (Poly-α, PfPK2, TA1, C3M69, C2M34 and M2490) for potential use in TES in Tanzania. These findings provide important information on these markers and parasite populations in the country and will facilitate future genomic studies and their application in TES and malaria surveillance.

## Methods

### Study sites

**Fig. 1:**
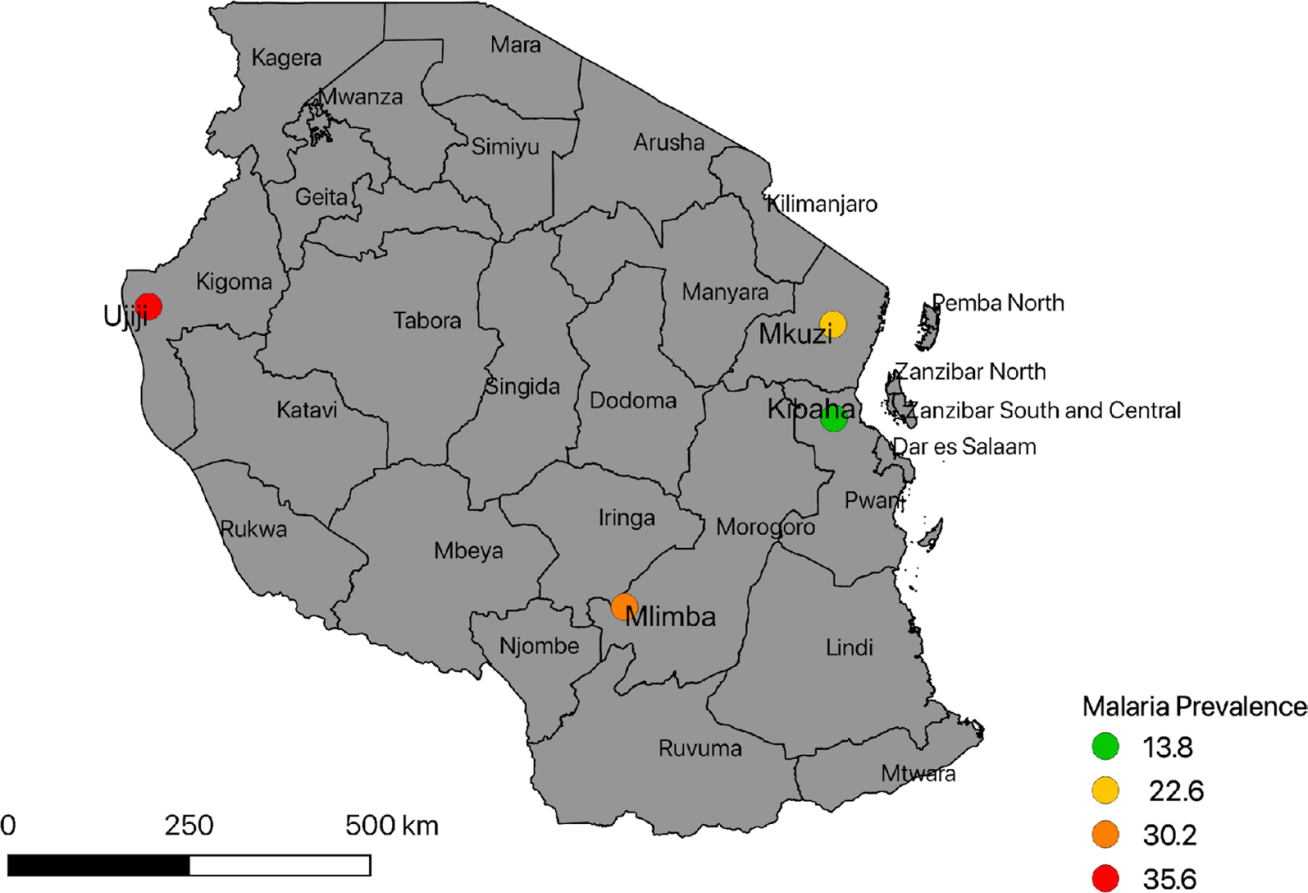
Map of Tanzania showing the four study sites of Kibaha, Mkuzi, Mlimba, and Ujiji. (Parasite prevalence data were obtained from the School Malaria Parasitological Survey of 2015 [38]

The samples used for this study were obtained from clinical malaria patients sampled in a TES that was conducted during and after the long rainy season between April and September 2016 [9]. It was undertaken in four geographically and epidemiologically distinct areas of Tanzania (Kibaha–Pwani, Mkuzi–Tanga, Mlimba–Morogoro and Ujiji–Kigoma), and these sites have been NMCP sentinel sites for monitoring anti-malarial efficacy since 1997 (Fig. 1) [23, 24]. The study sites were selected to represent distinct geographic areas of Tanzania. In Kibaha district of the Coastal region (Pwani), the study was conducted at Yombo Dispensary, which is located in an area that has transitioned from high to low malaria transmission (with a prevalence by rapid diagnostic tests for malaria (RDTs) in under-fives of < 10% in 2017) [31–33]. In Tanga region, the study site was Mkuzi Health Centre, which is located in Muheza district. Areas around Mkuzi have reported a progressive decline in malaria prevalence (in individuals aged < 20 years) from over 80% in the 1990s to < 10 in 2017 [34, 35]. Ujiji Health Centre is located in Kigoma urban district of Kigoma region. Parasite prevalence among under-fives (by RDTs) in Kigoma increased from 19.6% in 2007 to 38.1% in 2016, followed by a decrease to 24.4% in 2017; however, this was the highest prevalence in the country [31–33]. The fourth site of Mlimba Health Centre (parasite prevalence among under-fives in 2017 was < 10%) is located in Kilombero district of Morogoro region and has experienced a significant decline in malaria burden in the last two decades [36]. Additional details of the study sites were given elsewhere [9, 37].

### Study design and target population

Samples used for this analysis were collected during a single-arm prospective in vivo TES that assessed the therapeutic efficacy and safety of AL for the treatment of uncomplicated *Plasmodium falciparum* malaria and markers of artemisinin and lumefantrine resistance [9]. The study recruited 344 out of the 963 febrile children aged 6 months to 10 years who were screened according to the WHO protocol [25].

### Sample collection

Enrolled children were treated with AL and followed up for 28 days with clinical and parasitological assessments in the first 3 days post-treatment (days 1, 2 and 3) and once weekly from day 7 to 28 [9]. Thick and thin films were taken for the detection of malaria parasites during each visit. Dried blood spots (DBS) on filter papers were also collected at enrolment and from day 7 onward for molecular analyses of malaria parasites.

### Sample processing and genotyping

Parasite genomic DNA was extracted from DBS using QIAamp DNA mini-kits (Qiagen GmbH, Hilden, Germany) according to the manufacturer’s instructions. A single piece of each DBS sample was cut using a scissor, with sterilization between samples using ethanol to prevent contamination. The cut portion of DBS was used for DNA extraction and it contained an equivalent of three punches of 6 mm, with about 20–30 µL of blood. The extracted DNA was then eluted into 150 µL of elution buffer and used for different PCR analyses, without quantifying the amount of DNA. The results of different analyses by gel and capillary electrophoresis showed that the samples had DNA of sufficient quantity and quality as reported previously [9]. Genotyping of six neutral microsatellite markers was performed at the Centers for Disease Control and Prevention’s (CDC) Malaria Laboratory in Atlanta, USA. A total of 184 samples collected on day 0 and on the day of recurrent infections during TES were genotyped using 6 microsatellites and only day 0 samples (n = 94) were included in downstream analysis. These samples were analysed to distinguish recrudescent from new infections as previously reported [9] and to determine genetic diversity in the study populations. The microsatellite markers (TA1 on chromosome 6, Poly-α on chromosome 4, PfPK2 on chromosome 12, M2490 on chromosome 10, C2M34-313 on chromosome 2 and C3M69-383 on chromosome 3) were genotyped by nested PCR for all except C2M34-313 and C3M69-383 (which were analysed with a single-step PCR). Fragment size was measured by capillary electrophoresis on an ABI 3033 (Applied Biosystems) and scored using GeneMapper® Software Version 4.0 (Applied Biosystems) [39].

### Ethical considerations

Ethical clearance for the TES was obtained from the medical research coordinating committee (MRCC) of the National Institute for Medical Research (NIMR), while permission to conduct the study at the health facilities was sought in writing from the relevant regional and district medical authorities. Ethical clearance from the CDC was not required because the analysis of samples which was done at the CDC Malaria Laboratory, using samples without linked identifiers (de-identified samples), were determined by the CDC Center of Global Health’s Human Research Protection Coordinator to not constitute an engagement in human subjects’ research. Informed consent (oral and written) was obtained from parents or guardians before patients were screened to assess their eligibility for possible inclusion in the study.

### Data management and analysis

GeneScan chromatograms were analysed using GeneMapper® Software Version 4.0 (Applied Biosystems) with an internal size standard of 350 Rox. The stutter window was set to 2.5 for 2 bp repeats, 3.5 for 3 bp repeats and 4.5 for 4 bp repeats. The stutter ratio was set to 0.4 for the four markers, and for the remaining two markers (C2M34 and C3M69), a relatively higher stutter ratio (0.6) was set as they showed greater stuttering during manual inspection of the chromatograms. A cut-off of 1000 relative fluorescence units (RFUs) was used to distinguish true peaks from background signals for samples which produced more than one peak. All dominant peaks (i.e., those within the size range with the highest RFUs) and any additional alleles with a minimum height of 30% of the dominant allele were scored. All chromatograms were inspected manually to confirm call quality. Then, samples with low RFU density (10% of genotyped samples) were re-analysed with a minimum fluorescence of 200 RFU. Microsatellite haplotypes comprising more than 3 (50%) successfully typed markers were selected for further analysis.

For downstream population genetics analysis, multi-locus microsatellite allele data were converted into different formats using CONVERT software version 1.3.1. The number of genetically distinct parasite clones (multiplicity of infection, MOI) was calculated considering the maximum number of alleles detected at any of the six microsatellite loci. The number of clones for each population was determined by summing the total number of clones per isolate. The mean MOI for each population was calculated by dividing the total number of clones detected by the number of samples. Genetic diversity was measured by calculating allelic richness (*R*_*s*_) and expected heterozygosity (*H*_*e*_) using FSTAT software version 2.9.3.2 [40]. As a measure of inbreeding within populations (non-random association of alleles), the standardized index of association (*I*_A_^S^) was used to measure multi-locus linkage disequilibrium (LD) in each parasite population using LIAN version 3.6, applying a Monte Carlo test with 100,000 re-sampling steps [41].

STRUCTURE version 2.3.4 [42] was used to determine the number of population clusters (K) and whether the haplotypes clustered according to their geographic origin. The analysis was run 20 times for K = 1 to 20, and 100,000 Monte Carlo Markov Chain (MCMC) iterations after a burn-in period of 100,000, and using the admixture model. To obtain the optimal K value, the method of Evanno et al*.* [42] was used to calculate ΔK from the log probability of the data (LnP[D]) using STRUCTURE HARVESTER [43]. The STRUCTURE bar plots (ancestry coefficients) were visualized using the Cluster Markov Packager Across K (CLUMPAK) [44]. Genetic differentiation between populations was measured by calculating the *F*_ST_ statistic according to Nei [45]. Estimation of average heterozygosity and genetic distance from a small number of individuals was done using the pairwise.neifst function of the hierfstat R package [46]. The Mantel Test was performed to measure the associations between genetic distance and geographical distance between catchments using the mantel function of the R adegenet package [47]. To assess haplotype relatedness, the genetic distance metric (1 − pairwise allele sharing (*P*_*S*_)) was calculated and used to generate phylogenetic trees using the bionj Ape R package [48].

## Results

Among a total of 94 *Plasmodium falciparum* samples, 83 (88.3%) were successfully genotyped, and all gave positive results for 3 (50.0%) or more microsatellite markers (Table 1 and Additional file 1: Table S1). Only single infections or dominant haplotypes constructed from multiple infection data were included in downstream population genetic analyses (Additional file 1: Table S1). The number of clones per sample ranged from 1 to 4 (Additional file 2: Table S2), and a total of 38 (45.8%) (Additional file 3: Table S3) samples had single infections (samples with one allele at all 6 microsatellite loci), followed by samples carrying two distinct parasite clones (n = 32, 38.5%), three (n = 9, 10.84%), and only four samples carried four distinct clones (Additional file 2: Table S2). Overall, at least 38% of the samples in each population contained more than one parasite clone (polyclonal), and there was limited variability in the mean MOI among the populations (average MOI ranging from 1.68 to 1.88) (Table 1).

**Table 1:** Population genetic metrics of four Tanzanian *Plasmodium falciparum* populations

Population	Prevalence (%)^a^	N	n	*h*	*R*_*s*_ ± SD	*H*_*e*_ ± SD	*I*_A_^S^	MOI	Polyclonality (%)^b^
Kibaha	13.8	25	21	19	8.03 ± 2.8	0.85 ± 0.12	0.1569**	1.76	47.6
Ujiji-Kigoma	35.6	23	22	22	7.48 ± 2.1	0.84 ± 0.10	0.1736**	1.88	59.09
Mkuzi-Muheza	22.6	25	22	22	7.79 ± 2.4	0.82 ± 0.14	0.1095**	1.68	54.5
Mlimba-Kilombero	30.2	21	18	18	7.56 ± 2.3	0.80 ± 0.20	0.0903*	1.72	55.5

N: total number of samples; n: number of samples successfully genotyped; h: number of unique haplotypes; R_s_: Allelic richness; H_e_: expected heterozygosity; I_A_^S^: standard index of association as a measure of multi-locus linkage disequilibrium (LD); MOI: multiplicity of infection (mean number of clones per population); Polyclonality = proportion of samples containing more than one parasite clone

*p-value < 0.01, **p-value < 0.001

^a^Prevalence of malaria in the study districts in the 2015 school malaria parasitological survey [38]

^b^Polyclonality (%) refers to the proportion of infections with > 1 clones

### No significant difference in the mean multiplicity of infection was found among the populations

**Fig. 2:**
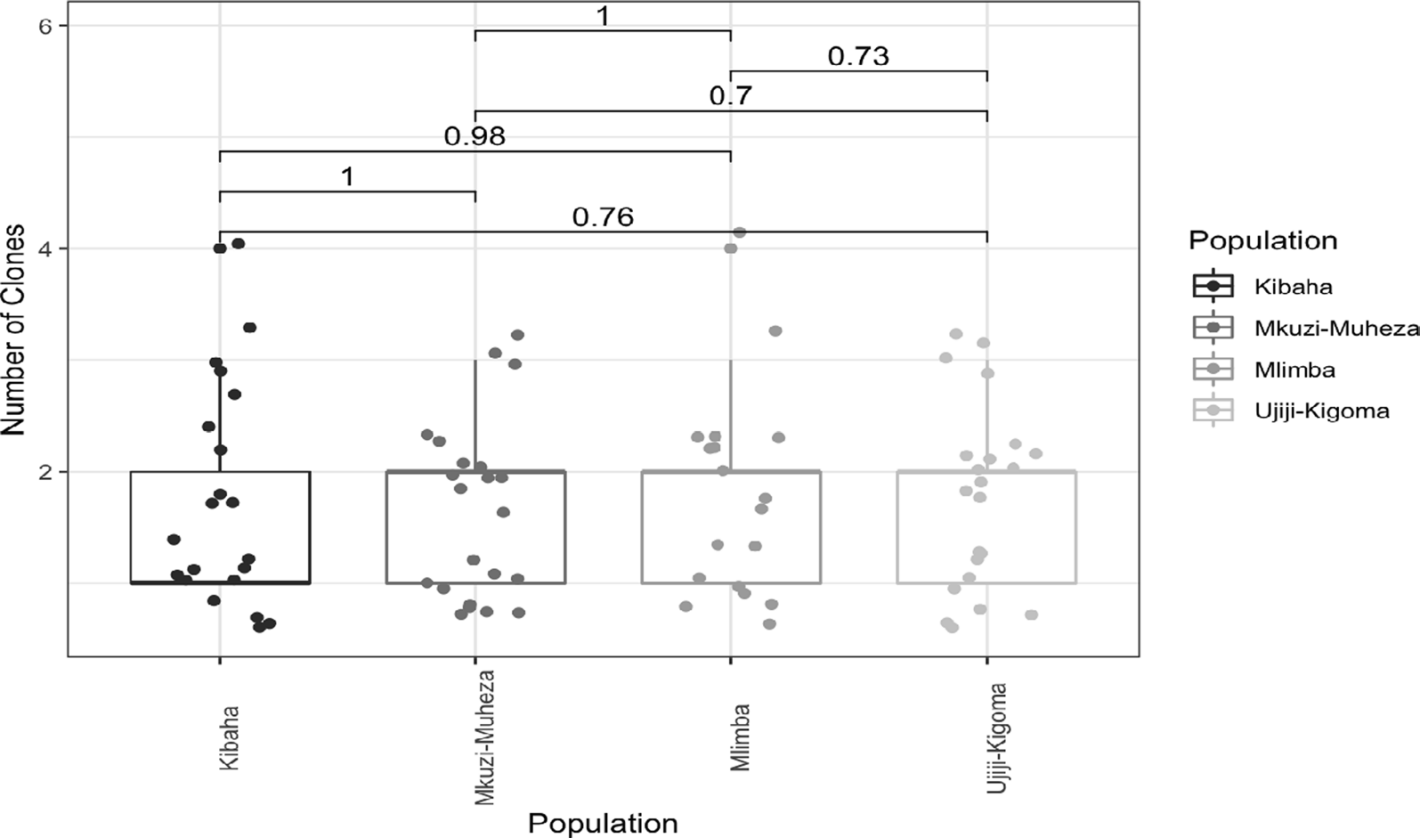
Multiplicity of infection in four Tanzanian *Plasmodium falciparum* populations. The Box and Whisker plots were generated from the number of clones determined for each microsatellite marker per population using R software. Dots indicate a haplotype, boxes indicate the interquartile range, the thick line indicates the median and the whiskers show the 95% confidence intervals. The numbers above the box plot indicate pairwise comparative p-values between populations, revealing a lack of significant difference in the MOI among the four sites

In malaria-endemic countries like Tanzania, individuals often carry more than one parasitic clone that is genetically different, referred to as the multiplicity of infection (MOI), also known as the complexity of infection (COI). The MOI occurs either due to repeated bites of infective mosquitoes or multiple clones in a single mosquito inoculum [49] and decreases with declining transmission. Here, we found a mean MOI of 1.73 across populations (range = 1–4 parasite clones per sample), and there was no statistically significant difference among the four populations (Kruskal‒Wallis test) (Fig. 2).

**Fig. 3:**
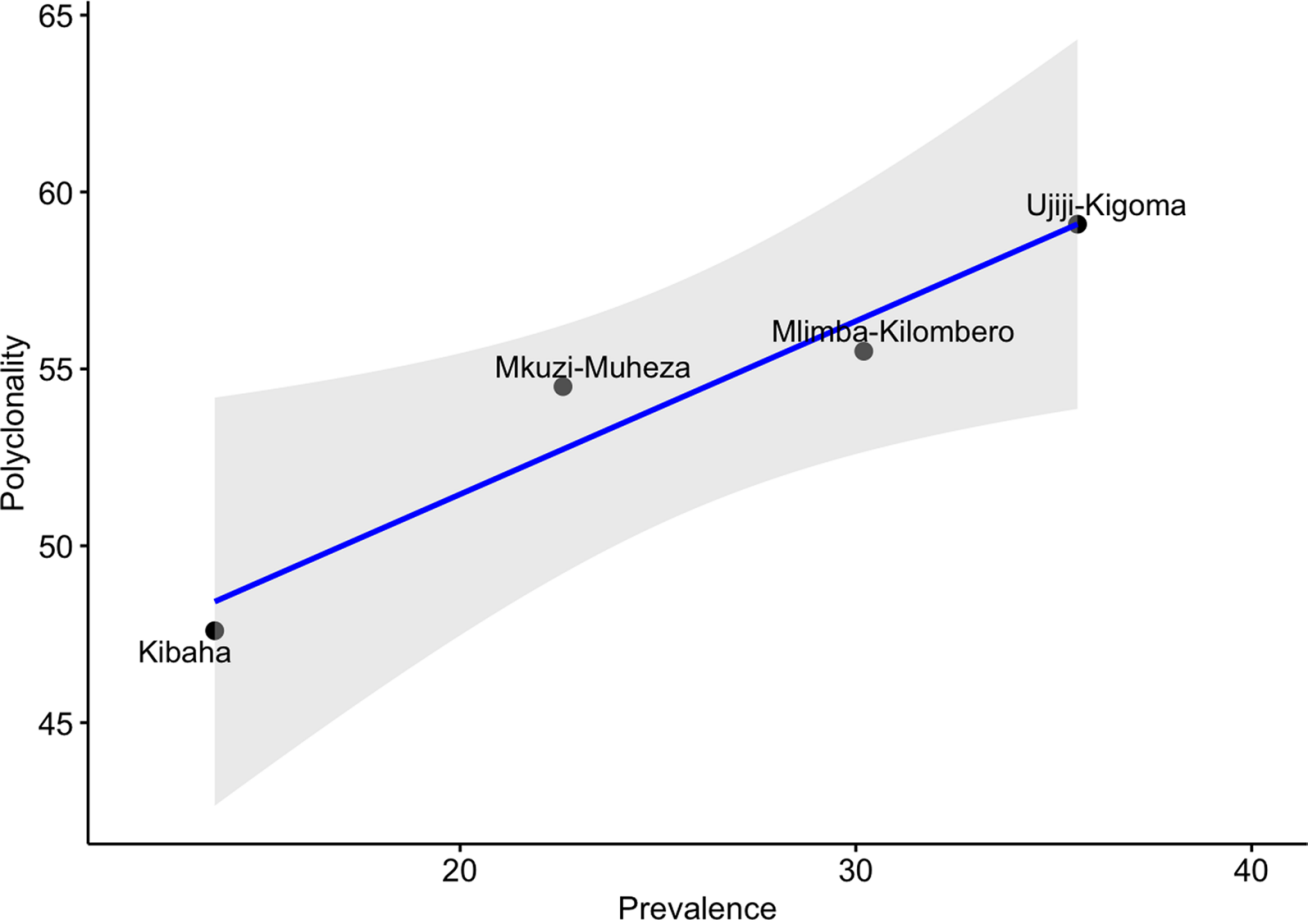
Association between parasite prevalence (by RDT) and the proportion of multiple infections (polyclonality) in four Tanzanian *Plasmodium falciparum* populations. The graph indicates a significant positive association between polyclonal infections and parasite prevalence across different geographic areas. Dots indicate population-level prevalence and proportion of polyclonal infections, the blue line indicates the Spearman Rank Correlation line, and the grey-shaded region represents the 95% confidence interval

The association between polyclonality (proportion of multiple infections) and parasite prevalence was assessed, and a positive correlation between polyclonality and malaria prevalence was observed (based on 2015 school survey data) per population (R = 0.97, *p-value* = 0.035, Spearman Rank Correlation) (Fig. 3). Polyclonality was lower in Kibaha and Muheza, which had a lower prevalence compared to Ujiji with higher polyclonality and prevalence of malaria.

### High genetic diversity but significant multi-locus linkage disequilibrium (LD)

**Fig. 4:**
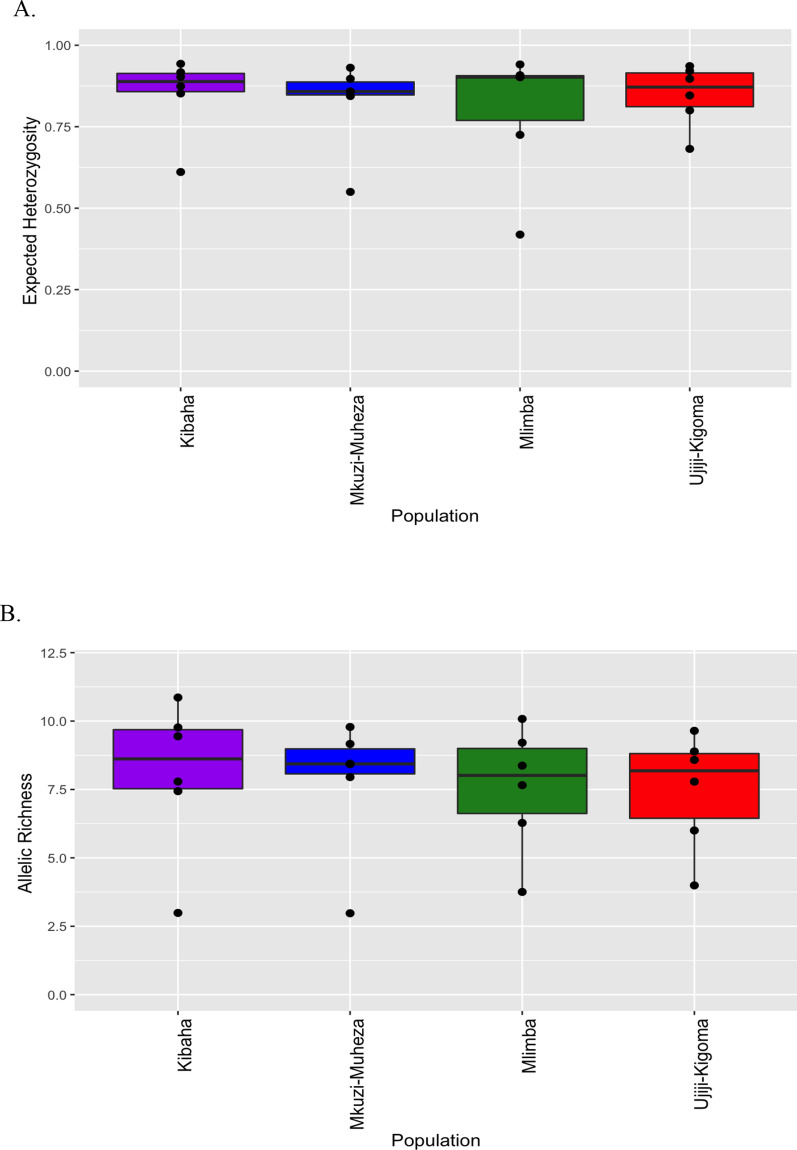
Genetic diversity [expected heterozygosity (**A**) and allelic richness (**B**)] of *Plasmodium falciparum* at the four geographic sites in Tanzania. The Box and Whisker plots were generated from the diversity metrics for each microsatellite marker per population using R software. Boxes indicate the interquartile range, the thick line indicates the median and the whiskers show the 95% confidence intervals

Of the 83 multi-locus haplotypes from successfully genotyped *Plasmodium falciparum* isolates, 53 (63.8%) were complete genotypes, 51 (96.2%) were unique, and only two haplotypes were identical to each other within Kibaha population. Regardless of transmission intensity, there was high genetic diversity of *Plasmodium falciparum*, with limited variability among the four parasite populations based on allelic richness (mean *R*_*S*_ = 7.27, range = 7.48–8.03, for an adjusted minimum sample size of 18 per site) and expected heterozygosity (mean *H*_*e*_ = 0.83, range = 0.80–0.85) (Table 1, Fig. 4). However, according to the Index of Association (*I*_*A*_^*S*^) analysis, which is a measure of multi-locus LD (which emerges when genotypes are related), all the parasite populations from the four sites showed significant multi-locus LD (Table 1). This could be explained by the presence of some degree of inbreeding despite high transmission intensity in some areas.

**Fig. 5:**
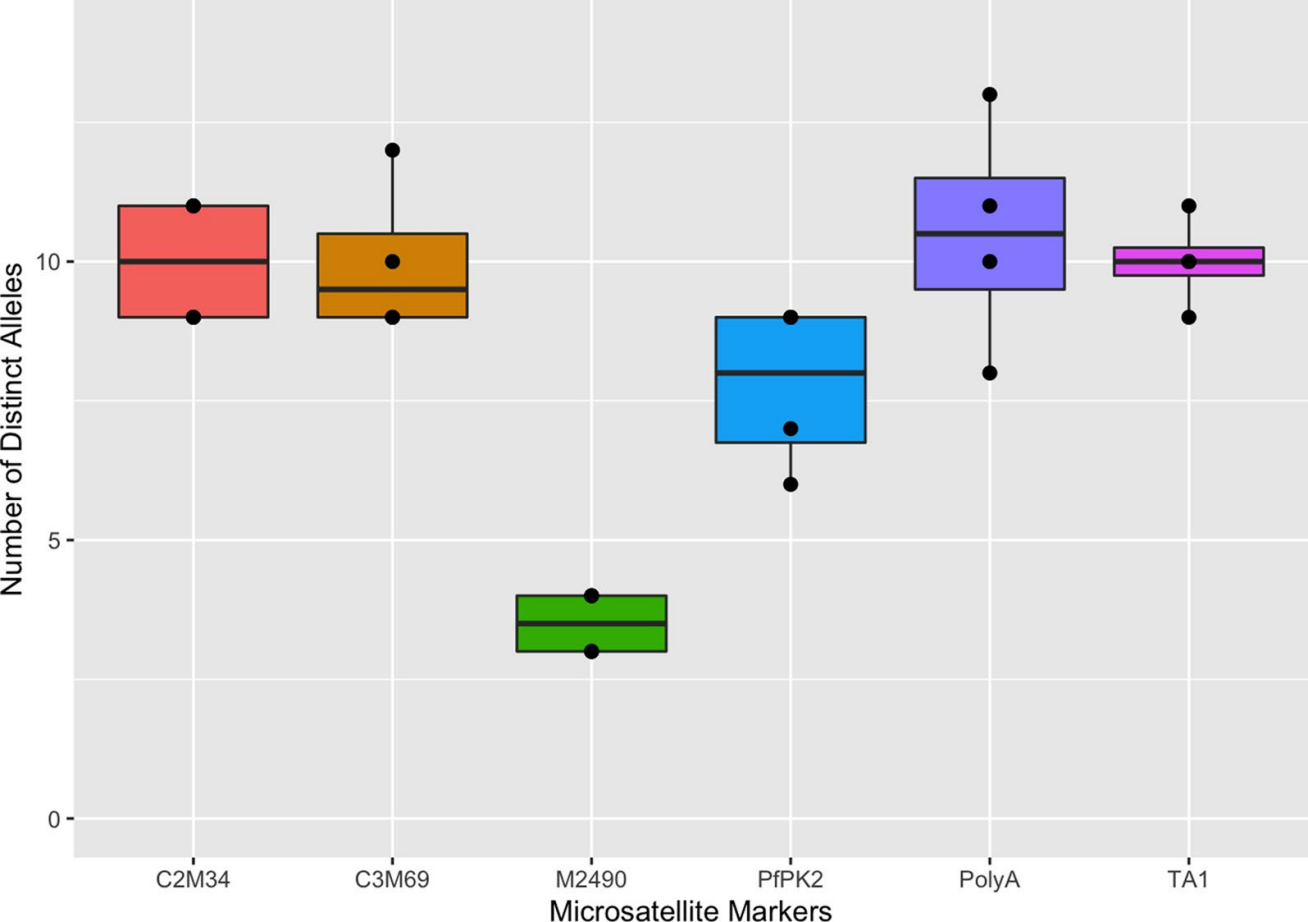
Diversity of *Plasmodium falciparum* microsatellite markers among parasites from the four sites in Tanzania. The Box and Whisker plots were generated from unique allele counts for each microsatellite marker using R software. Boxes indicate the interquartile range, the thick line indicates the median and the whiskers show the 95% confidence intervals

Furthermore, the diversity of microsatellite markers was assessed, and there was high variability in the alleles present per marker (A = 3–13) (Fig. 5), with variable frequencies for the four different sites. The marker M2490 was the least diverse microsatellite, with only 3.5 mean number of distinct alleles detected across the four populations, while Poly-α was the most diverse microsatellite marker (A = 13, *H*_*e*_ = 0.91), followed by C2M34 (A = 11, *H*_*e*_ = 0.89). These two highly polymorphic markers (poly-α and C2M34), together with C3M69 and TA1, can be used for the detection of parasite clones in Tanzania.

### Lack of population structure and genetic differentiation

**Fig. 6:**
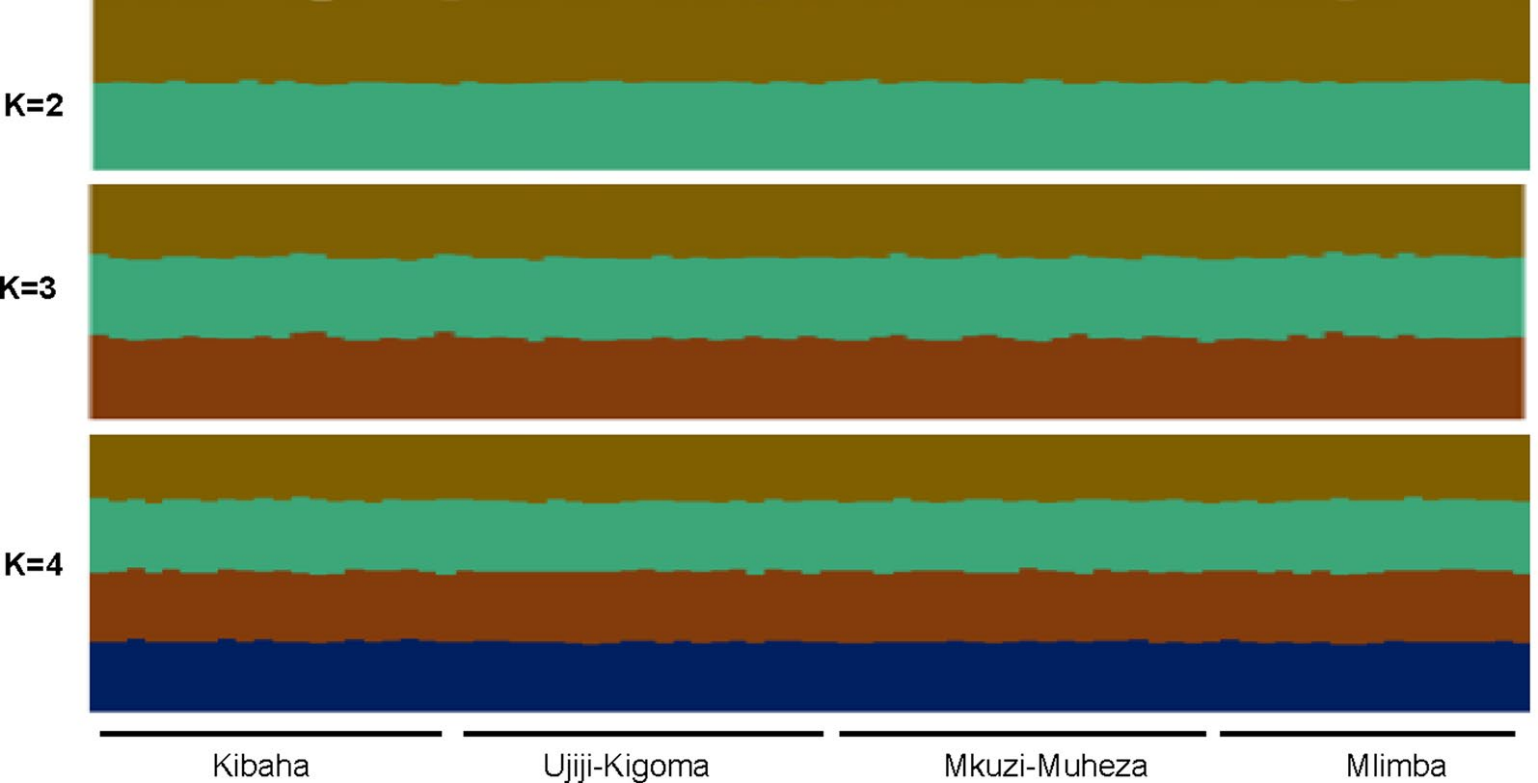
Bayesian cluster analysis of *Plasmodium falciparum* microsatellite haplotypes from the four sites of Tanzania. Structure bar plots representing individual ancestry coefficients are shown for K = 2, 3 and 4, and each vertical bar represents an individual genotype and the membership coefficient (Q) within each of the genetic populations, as defined by the different colours

To investigate the presence of parasite population structure among the four Tanzanian sites, cluster analysis of the haplotypes was conducted using STRUCTURE version 2.3.4. No evidence of any population structure was detected from K = 2–4, and the ancestry of the genotypes was equally split between the genetic populations, revealing no evidence of population structure (Fig. 6).

**Fig. 7:**
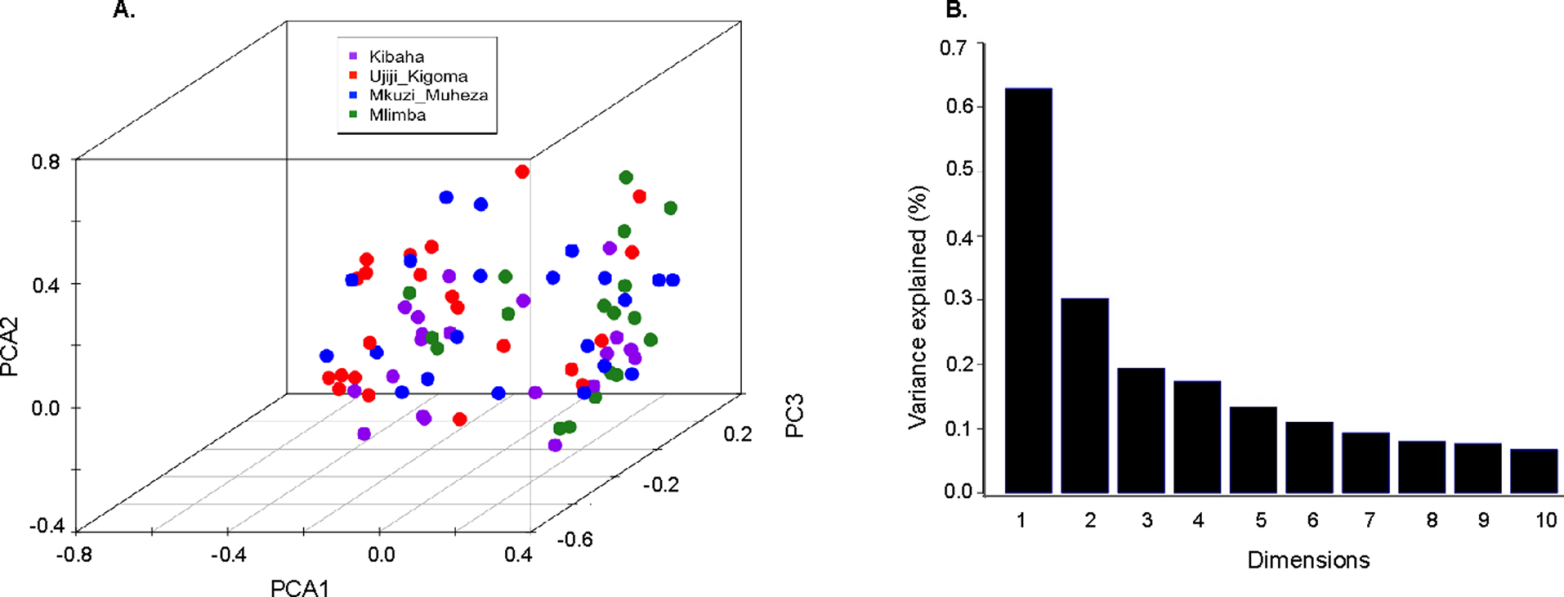
*Plasmodium falciparum* haplotype clustering. **A** Principal component analysis (PCA) of *Plasmodium falciparum* haplotypes. Dots indicate individual microsatellite haplotypes, and colours indicate the four sample collection sites. **B** The percentage of variance explained by each principal component (PC)

Further cluster analysis of the haplotypes using principal component analysis (PCA, performed with the princomp function in the R package) also revealed no signatures of population structure and no clustering of isolates according to geographic origin (Fig. 7).

### Gene flow and population connectivity

To assess gene flow and population connectivity, pairwise genetic differences among the four parasite populations were calculated based on Jost’s D metric [50] and FST according to Nei [45] using the pairwise. neifst function available in the hierfstat R package. Very low levels of genetic differentiation were observed between populations, confirming that *Plasmodium falciparum* populations from these sites are highly panmictic (Table 2). Mantel test was also conducted to assess the correlation between pairwise genetic distance and pairwise geographic distance in km as an indication of gene flow and parasite connectivity. The differentiation of parasite populations was not significantly associated with geographical distance between populations and therefore did not fit the Isolation-by-Distance model (Mantel statistic r = 0.072, *p-value* = 0.59).

**Table 2:** Pairwise genetic differentiation among parasite populations in Tanzania

	Kibaha	Ujiji-Kigoma	Mkuzi-Muheza	Mlimba
Kibaha		0.01	0.009	0.038
Ujiji-Kigoma	0.37		0.005	0.001
Mkuzi-Muheza	0.22	0.14		0.003
Mlimba	0.34	0.37	0.24	

Top right = pairwise *F*_*ST*_, left bottom = pairwise Jost’s D

### Haplotype relatedness

**Fig. 8:**
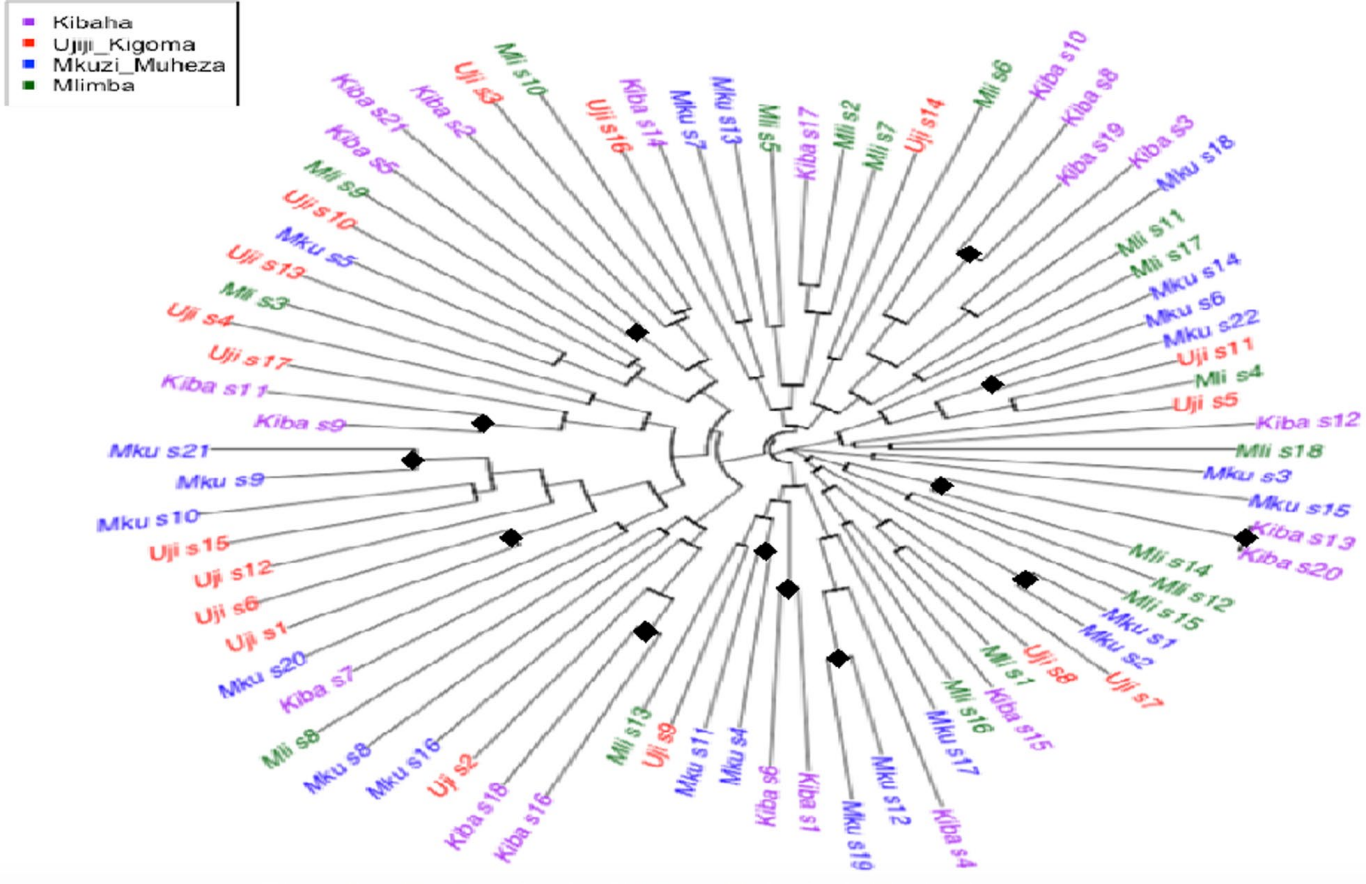
Relatedness of *Plasmodium falciparum* haplotypes in Tanzania. Neighbour-Joining tree showing low levels of similarity of the multi-locus *Plasmodium falciparum* haplotypes between most isolates with similar haplotypes within populations. Tips of the NJ tree are colour-coded according to the four geographic sites, and black diamonds indicate bootstrap values > 50

To assess relatedness in *Plasmodium falciparum*, pairwise comparisons among all the isolates were conducted using the dist.gene command in the R Ape package. The results showed that, on average, the majority of the isolates had only one identical allele among all six markers, and only a few isolates shared more than 50% of the alleles (three or more alleles). Phylogenetic analysis using neighbour-joining tree also confirmed a lack of population structure and geographic clustering of the genotypes. However, more haplotypes were clustering together within than between populations (Fig. 8).

## Discussion

This study included samples from four geographically distinct parasite populations (located 296 to 1211 km apart) from areas with different transmission intensities to assess polymorphisms and genetic diversity of *Plasmodium falciparum’s* six neutral microsatellite markers (Poly-α, PfPK2, TA1, C3M69, C2M34 and M2490) for potential use in TES in Tanzania. It also aimed to capture the spatial genetic diversity and population structure of Tanzanian *Plasmodium falciparum*. The findings showed that four markers (Poly-α, C2M34, C3M69 and TA1) had high diversity and could be adopted as validated markers for use in TES in Tanzania. As recently recommended by WHO [29] and a previous study that showed that a combination of four microsatellite markers with sufficient diversity is required in TES [51, 52], these microsatellite markers can be included in the revised workflow for TES in Tanzania. The new panel should replace the old protocol based on genotyping of *msp1, msp2* and *glurp* for distinguishing recrudescent from new infections in ongoing TES in Tanzania. However, the areas around TES sites have increasingly reported a decline and heterogeneity of malaria transmission in the past two decades, suggesting that continuous assessment of these and possibly other microsatellite markers will be critical. This approach will ensure that high-resolution markers are used and that the efficacy of anti-malarials is not underestimated due to limited discrimination power of the markers. Additional methods such as targeted amplicon sequencing can also be explored based on the capacity of the laboratory, as recently recommended [53].

This study also showed high diversity, a lack of population structure and a high level of polyclonality despite the varying prevalence of malaria among the study sites. The results suggest that these areas still have high malaria transmission rates, but there is little evidence of impact of interventions deployed by NMCP on transmission dynamics. However, a significant correlation between parasite prevalence and polyclonality (as a proxy of malaria transmission intensity) was detected as expected, given that in areas with higher malaria prevalence, humans are exposed to multiple mosquito bites (superinfection) or infections with multiple clones (co-transmission) [54, 55]. A strong correlation between parasite prevalence and polyclonality has been reported in other studies [56–58] and needs to be monitored as a surrogate measure of potential changes in malaria transmission due to the impacts of interventions. In Papua New Guinea (PNG), the *Plasmodium falciparum* MOI was associated with parasite prevalence, but the diversity of the polymorphic marker sizes remained high despite wide variation in prevalence at different sites [56–58]. In contrast to these findings, a study in Indonesia [59] reported lower genetic diversity, which was consistent with the low level of malaria transmission at the study sites and could be a result of longer-term sustained low transmission in this area than in PNG and Tanzania.

Microsatellites are highly polymorphic and rapidly evolving; therefore, long-term sustained low transmission may be needed to detect signals of low diversity [30]. In PNG, studies have followed *Plasmodium falciparum* populations in terms of declining transmission for more than 9 years and reported very minor changes in microsatellite diversity [60]. Moreover, high transmission intensity, high polyclonality and, therefore, high rates of recombination between distinct clones (outcrossing) might obscure the expected association between the MOI and transmission intensity (prevalence) in different transmission zones. However, in low-transmission areas such as South America, studies conducted in Ecuador and Peru have reported infections containing clonal parasites with clear population structures [61, 62].

In addition to the MOI as a proxy for transmission intensity, estimating the extent of parasite genetic diversity and population structure is essential for obtaining a deeper understanding of malaria epidemiology and transmission dynamics as well as evaluating the impact of malaria control interventions [63]. Polymorphic markers can also be used for the detection of different parasite clones in different studies including TES. In this study, it was shown that parasite genetic diversity was high at the four Tanzanian sites regardless of the prevalence of infection and that the respective parasite populations appeared to be highly mixed with no clear genetic structure according to geographic origin. Thus, the high polymorphism at all sites and with all markers suggests that these markers (especially the four topmost) can sufficiently be used in TES to distinguish recrudescent from new infections as recommended by the WHO [29].

Unlike the expectation that geographical isolation causes limited migration among subpopulations and geographical population structure, there was no significant genetic differentiation (measured by *F*_ST_) between distant and nearby parasite populations. These results suggest high malaria transmission intensity and/or extensive parasite migration as well as mixing throughout the country despite significant improvements in malaria control strategies and drastic declines in malaria transmission and disease burden in recent years. These findings support previous observations where genetic diversity, geographic clustering and inbreeding with strong LD as population genetic signals are expected in low-transmission areas, whereas high proportions of polyclonal infections, high diversity and panmictic parasite populations are expected in areas with high levels of transmission [30]. Generally, the levels of allelic diversity, parasite outcrossing, and gene flow are high in African populations, low in South American populations, and intermediate in Southeast Asian populations [30]. The results of this study support the situation of continuing highly endemic transmission dynamics in the country despite the expected substantial impact of recent interventions on parasite prevalence in Tanzania. The observed differences between this and recent studies, which were conducted in Tanzania and showed population structure among parasites from different parts of the country [64, 65], could be due to the markers used; SNPs and WGS data compared to microsatellites used in the current study. Validation of microsatellite markers for surveillance is important because they have been the gold standard tool for determining the genetics of malaria parasite populations for many years. Furthermore, they are cheaper and easier to access for resource-limited laboratories. Ongoing and future studies will test different markers to increase the resolution and robustness of capturing different population genetics metrics that will be useful in assessing the impact of interventions and progress toward malaria elimination in Tanzania. Additionally, optimization of markers for molecular genotyping of samples collected in TES needs to be pursued as recently recommended [53].

In contrast to the above findings, there was significant multi-locus LD within populations, suggesting some level of inbreeding of related parasites and repeated haplotypes, indicating the occurrence of some clonal transmission (monoclonal infections transmitted by the mosquito vector in which parasite sexual recombination occurred between genetically identical clones, albeit within the limitations of the markers used). An additional explanation for this finding could be due to the presence of subpopulations within populations (Wahlund effect) [66], as the samples for this study were obtained from clinical sites where patients usually come from different geographic areas to seek medical care. Other studies in different malaria-endemic countries found similar results, and significant LD despite the high genetic diversity and high proportion of polyclonal infections caused by *Plasmodium falciparum* [67, 68] and *Plasmodium vivax* [69, 70]. The detection of significant LD has important implications that could facilitate inbreeding and dispersal of multi-locus drug resistance haplotypes or other virulent strains. As transmission decreases in Tanzania due to intensive control activities, as shown elsewhere [60], the presence of LD combined with a lack of geographic population structure is highly likely to facilitate such events, and it could be a future challenge in achieving malaria elimination.

There was high diversity in each of the microsatellite markers, indicating that a few highly polymorphic markers (Poly-α and C2M34) can be used to track the MOI of *Plasmodium falciparum* in Tanzania. However, PfPK2 which was recommended by WHO together with Poly-α was less polymorphic and less informative suggesting that these markers need to be optimized to fit the local malarial epidemiology before they can be adopted for use in TES. In addition, the genotyped markers may have limited the resolution of the population structure. The microsatellite panel used had few markers (only six, with less than one per chromosome); many were highly polymorphic (many alleles), and they were prone to technical artefacts [71]. In addition, the sample size per population was relatively limited, with approximately 20 samples successfully genotyped per site. Therefore, subtle differences between populations may not be detected. For example, in Kibaha, the same haplotype was found in two samples, whilst in all the other populations, all the haplotypes were unique. If more samples had been genotyped, additional repeated haplotypes may have been found, and diversity measures altered somewhat. Further analysis of large numbers of samples (n > 50) from additional sites (again with varying transmission intensity) and utilizing larger numbers of highly polymorphic microsatellite markers [30, 72, 73] and/or comparing them with SNP barcodes [74], amplicons [75–78] and WGS [79] will be required to optimize these markers. This should also be part of the ongoing initiatives to establish a molecular surveillance platform to support policy and decision-making by the Tanzanian NMCP in their strategy to eliminate malaria by 2030. Nevertheless, the data generated provide findings of useful markers for TES and parasite populations in Tanzania showing that there are potentially large, diverse and highly intermixing parasites despite strong reductions in infection prevalence and disease burden. The findings also provide useful baseline information for future monitoring of parasite populations in response to the ongoing malaria interventions.

## Conclusion

Microsatellite genotyping revealed high polyclonality and genetic diversity but without any significant population structure. Poly-α, C2M34, C3M69 and TA1 were the topmost polymorphic markers and Poly-α alone or with any of the other three markers could be adopted for use in TES in Tanzania. Failure to reveal any significant population structure among parasite populations could be due to high transmission or inherent limitations of small numbers of microsatellite markers and sample size. More studies covering sites with varying transmission intensities, more samples and using other genotyping markers will be required for establishing an effective molecular surveillance system to support the implementation of TES and area-specific interventions in Tanzania and for monitoring the impacts of the current and future malaria interventions.

### Supplementary Information

**Additional file 1: Table S1.** Microsatellite final data set used for downstream analysis. The numbers indicate actual allele calls and ? indicate no call made. Only the dominant allele shown here when samples have more than allele calls per marker.

**Additional file 2: Table S2.** Number of allele calls per marker for each sample. The numbers indicate the actual total number of alleles called and 0 indicate no call made. Maximum number of allele call used to determine multiplicity of infection (MOI).

**Additional file 3: Table S3.** List of haplotypes for samples with one allele at all 6 microsatellite loci. The numbers indicate actual allele calls and ? indicate no call made. Multiplicity of infection = MOI.

## Data Availability

The microsatellite dataset used for this study is available and has been submitted with this paper as Additional file 1: Table S1. Additional data including the prevalence of malaria in the four sites can be obtained from the NMCP upon making an official request.

## Abbreviations

ACT: Artemisinin-based combination therapy
AL: Artemether–lumefantrine
CDC: Centers for Disease Control and Prevention
DBS: Dried blood spot
DNA: Deoxyribonucleic acid
MRCC: Medical Research Coordinating Committee of NIMR
PARMA: Partnership for Antimalarial Resistance Monitoring in Africa network
PMI: U.S President’s Malaria Initiative
NIMR: National Institute for Medical Research
NMCP: National Malaria Control Programme
PCR: Polymerase chain reaction
SNP: Single nucleotide polymorphism
TES: Therapeutic efficacy study
WHO: World Health Organization
