# Supplementary material for: Microsatellites reveal high polymorphism and high potential for use in anti-malarial efficacy studies in areas with different transmission intensities in mainland Tanzania
Source: Malar J. 2024 Mar 15;23:79. doi: 10.1186/s12936-024-04901-6 (PMC10943981; doi:10.1186/s12936-024-04901-6)
Supplement: Supplementary file 1 — Additional file 1: Table S1. Microsatellite final data set used for downstream analysis. The numbers indicate actual allele calls and ? indicate no call made. Only the dominant allele shown here when samples have more than allele calls per marker. [file 12936_2024_4901_MOESM1_ESM.docx]

**Table S1: Microsatellite final data set used for downstream analysis. The numbers indicate actual allele calls and ? indicate no call made. Only the dominant allele shown here when samples have more than allele calls per marker.**

| **Sample_id** | **Population** | **PolyA** | **M2490** | **PfPK2** | **TA1** | **C2M34** | **C3M69** | MOI |
| --- | --- | --- | --- | --- | --- | --- | --- | --- |
| T0301D0 | Kibaha | 164 | 83 | 174 | 165 | 222 | 151 | 1 |
| T0305D0 | Kibaha | 149 | 79 | 180 | 163 | 218 | 147 | 2 |
| T0307D0 | Kibaha | 155 | 79 | 165 | ? | 222 | 131 | 1 |
| T0313D0 | Kibaha | 149 | ? | 168 | 163 | 222 | ? | 1 |
| T0316D0 | Kibaha | 167 | 79 | 168 | 173 | 218 | ? | 2 |
| T0321D0 | Kibaha | 164 | 83 | 174 | 183 | 238 | 137 | 2 |
| T0322D0 | Kibaha | 146 | 79 | 171 | 189 | 230 | 123 | 1 |
| T0324D0 | Kibaha | 152 | 71 | 165 | 167 | 222 | 131 | 4 |
| T0325D0 | Kibaha | 161 | 83 | 180 | ? | 218 | 123 | 1 |
| T0332D0 | Kibaha | 155 | 71 | 165 | 167 | 250 | 133 | 2 |
| T0341D0 | Kibaha | 161 | 79 | 180 | ? | 218 | 143 | 4 |
| T0342D0 | Kibaha | 158 | 83 | 180 | 193 | 230 | 169 | 2 |
| T0346D0 | Kibaha | 179 | 83 | 177 | 175 | 234 | 161 | 1 |
| T0348D0 | Kibaha | 140 | 83 | 165 | ? | 250 | 137 | 1 |
| T0350D0 | Kibaha | 167 | 83 | 159 | 163 | 258 | 123 | 1 |
| T0355D0 | Kibaha | 152 | 71 | 168 | 163 | 218 | 139 | 3 |
| T0358D0 | Kibaha | 146 | 83 | 162 | 183 | 222 | 143 | 1 |
| T0360D0 | Kibaha | ? | 71 | 165 | 169 | 218 | 147 | 3 |
| T0364D0 | Kibaha | 161 | 83 | 165 | 167 | 222 | 131 | 1 |
| T0368D0 | Kibaha | 179 | 83 | 177 | 175 | 234 | 161 | 1 |
| T0369D0 | Kibaha | 167 | 79 | 174 | 177 | 230 | 125 | 2 |
| T0402D0 | Ujiji-Kigoma | 143 | ? | ? | 189 | 238 | 123 | 2 |
| T0411D0 | Ujiji-Kigoma | 152 | ? | 162 | 169 | 218 | 129 | 2 |
| T0412D0 | Ujiji-Kigoma | 149 | 79 | ? | 165 | 250 | 139 | 1 |
| T0418D0 | Ujiji-Kigoma | 161 | ? | ? | ? | 254 | 137 | 1 |
| T0421D0 | Ujiji-Kigoma | 161 | 87 | 171 | 167 | 254 | 137 | 1 |
| T0424D0 | Ujiji-Kigoma | 140 | 83 | 168 | 183 | 230 | 123 | 2 |
| T0428D0 | Ujiji-Kigoma | 173 | ? | ? | 189 | 222 | 143 | 1 |
| T0430D0 | Ujiji-Kigoma | 161 | ? | ? | ? | 242 | 135 | 1 |
| T0436D0 | Ujiji-Kigoma | 143 | 71 | 162 | 177 | 226 | 123 | 3 |
| T0439D0 | Ujiji-Kigoma | 140 | 83 | 162 | 173 | 226 | 123 | 3 |
| T0441D0 | Ujiji-Kigoma | 149 | 83 | ? | 169 | 218 | 137 | 1 |
| T0444D0 | Ujiji-Kigoma | 146 | ? | ? | ? | 218 | 143 | 1 |
| T0447D0 | Ujiji-Kigoma | 161 | 79 | 162 | 179 | 242 | 137 | 2 |
| T0448D0 | Ujiji-Kigoma | 143 | 83 | 168 | 179 | 218 | 135 | 2 |
| T0454D0 | Ujiji-Kigoma | 149 | ? | ? | ? | 210 | 123 | 2 |
| T0460D0 | Ujiji-Kigoma | 149 | ? | 150 | ? | 230 | 123 | 3 |
| T0466D0 | Ujiji-Kigoma | 140 | 79 | 162 | 169 | 222 | 127 | 2 |
| T0468D0 | Ujiji-Kigoma | 155 | 83 | 165 | 163 | 246 | 163 | 1 |
| T0470D0 | Ujiji-Kigoma | 179 | ? | ? | ? | 218 | 123 | 2 |
| T0474D0 | Ujiji-Kigoma | 155 | ? | ? | ? | 230 | 139 | 1 |
| T0475D0 | Ujiji-Kigoma | 143 | 83 | 186 | ? | 266 | 143 | 2 |
| T0485D0 | Ujiji-Kigoma | 161 | 79 | ? | ? | 226 | 123 | 4 |
| T0701D0 | Mkuzi-Muheza | 152 | 83 | 189 | 163 | 218 | 123 | 1 |
| T0702D0 | Mkuzi-Muheza | 152 | 83 | 189 | 163 | 218 | 123 | 1 |
| T0704D0 | Mkuzi-Muheza | 173 | 83 | ? | 133 | 242 | 123 | 2 |
| T0706D0 | Mkuzi-Muheza | 149 | 83 | 174 | 167 | 270 | 123 | 2 |
| T0707D0 | Mkuzi-Muheza | 140 | 79 | 162 | ? | ? | 123 | 2 |
| T0708D0 | Mkuzi-Muheza | 143 | 83 | 162 | 183 | ? | 135 | 1 |
| T0709D0 | Mkuzi-Muheza | 146 | 83 | 165 | ? | 218 | 139 | 2 |
| T0710D0 | Mkuzi-Muheza | 164 | 71 | 159 | 169 | 238 | 149 | 2 |
| T0711D0 | Mkuzi-Muheza | 146 | ? | ? | ? | 218 | 123 | 3 |
| T0714D0 | Mkuzi-Muheza | 143 | ? | ? | ? | 254 | 161 | 1 |
| T0717D0 | Mkuzi-Muheza | 149 | 83 | 177 | 167 | 222 | 137 | 1 |
| T0718D0 | Mkuzi-Muheza | 149 | 71 | 162 | 163 | 222 | 123 | 3 |
| T0734D0 | Mkuzi-Muheza | 161 | 83 | 168 | 163 | 226 | 143 | 2 |
| T0743D0 | Mkuzi-Muheza | 167 | 83 | 171 | 183 | 246 | 135 | 1 |
| T0747D0 | Mkuzi-Muheza | 173 | 83 | 192 | 165 | 246 | 149 | 2 |
| T0752D0 | Mkuzi-Muheza | 158 | 71 | 171 | 171 | 218 | 143 | 1 |
| T0756D0 | Mkuzi-Muheza | 149 | 83 | 162 | 183 | 222 | 153 | 1 |
| T0758D0 | Mkuzi-Muheza | 155 | 79 | ? | 173 | 222 | 135 | 1 |
| T0761DO | Mkuzi-Muheza | 140 | 71 | 162 | 163 | 222 | 121 | 2 |
| T0767D0 | Mkuzi-Muheza | 146 | 71 | ? | 187 | 262 | 123 | 1 |
| T0771D0 | Mkuzi-Muheza | 167 | ? | ? | ? | 218 | 155 | 2 |
| T0774D0 | Mkuzi-Muheza | 143 | 83 | 162 | 177 | 238 | 135 | 3 |
| T08002D0 | Mlimba-Kilombero | 149 | 83 | 162 | 163 | 226 | 123 | 1 |
| T08008D0 | Mlimba-Kilombero | 146 | 83 | 165 | 197 | 222 | 143 | 1 |
| T08014D0 | Mlimba-Kilombero | 161 | ? | 171 | 191 | 218 | 123 | 2 |
| T08031D0 | Mlimba-Kilombero | 143 | 83 | 168 | 183 | 230 | 135 | 2 |
| T08032D0 | Mlimba-Kilombero | 161 | 83 | 171 | 173 | 234 | 143 | 1 |
| T0803D0 | Mlimba-Kilombero | 155 | 87 | 162 | 173 | 246 | 141 | 1 |
| T0808D0 | Mlimba-Kilombero | 146 | 83 | 162 | 173 | 262 | 143 | 1 |
| T0811D0 | Mlimba-Kilombero | 131 | 71 | 153 | 149 | 242 | 141 | 2 |
| T0833D0 | Mlimba-Kilombero | 182 | 79 | 162 | 183 | 234 | 133 | 2 |
| T0834D0 | Mlimba-Kilombero | 149 | 79 | 195 | 179 | 246 | 143 | 2 |
| T0836D0 | Mlimba-Kilombero | 143 | 83 | 171 | 173 | 222 | 145 | 1 |
| T08424D0 | Mlimba-Kilombero | 158 | 83 | 162 | 167 | 214 | 145 | 1 |
| T0848D0 | Mlimba-Kilombero | 149 | 83 | 162 | 173 | 238 | 137 | 3 |
| T0852D0 | Mlimba-Kilombero | 143 | 83 | 162 | 175 | 234 | 123 | 4 |
| T0854D0 | Mlimba-Kilombero | 158 | 83 | 162 | 175 | 218 | 131 | 2 |
| T0855D0 | Mlimba-Kilombero | 155 | 83 | 162 | 179 | 258 | 123 | 1 |
| T0874D0 | Mlimba-Kilombero | 155 | 83 | 171 | 167 | 222 | 147 | 2 |
| T0880D0 | Mlimba-Kilombero | 155 | 83 | 159 | 181 | 230 | 135 | 2 |
