# Supplementary material for: Microsatellites reveal high polymorphism and high potential for use in anti-malarial efficacy studies in areas with different transmission intensities in mainland Tanzania
Source: Malar J. 2024 Mar 15;23:79. doi: 10.1186/s12936-024-04901-6 (PMC10943981; doi:10.1186/s12936-024-04901-6)
Supplement: Supplementary file 2 — Additional file 2: Table S2. Number of allele calls per marker for each sample. The numbers indicate the actual total number of alleles called and 0 indicate no call made. Maximum number of allele call used to determine multiplicity of infection (MOI). [file 12936_2024_4901_MOESM2_ESM.docx]

**Table S2: Number of allele calls per marker for each sample. The numbers indicate the actual total number of alleles called and 0 indicate no call made. Maximum number of allele call used to determine multiplicity of infection (MOI)**

| **Sample_id** | **Population** | **PolyA** | **M2490** | **PfPK2** | **TA1** | **C2M34** | **C3M69** | **MOI** |
| --- | --- | --- | --- | --- | --- | --- | --- | --- |
| T0301D0 | Kibaha | 1 | 1 | 1 | 1 | 1 | 1 | 1 |
| T0305D0 | Kibaha | 2 | 2 | 1 | 1 | 1 | 1 | 2 |
| T0307D0 | Kibaha | 1 | 1 | 1 | 0 | 1 | 1 | 1 |
| T0313D0 | Kibaha | 1 | 0 | 1 | 1 | 1 | 0 | 1 |
| T0316D0 | Kibaha | 1 | 1 | 2 | 1 | 1 | 0 | 2 |
| T0321D0 | Kibaha | 1 | 1 | 2 | 1 | 1 | 1 | 2 |
| T0322D0 | Kibaha | 1 | 1 | 1 | 1 | 1 | 1 | 1 |
| T0324D0 | Kibaha | 4 | 2 | 2 | 2 | 1 | 1 | 4 |
| T0325D0 | Kibaha | 1 | 1 | 1 | 0 | 1 | 1 | 1 |
| T0332D0 | Kibaha | 1 | 2 | 2 | 1 | 1 | 1 | 2 |
| T0341D0 | Kibaha | 1 | 2 | 4 | 1 | 1 | 1 | 4 |
| T0342D0 | Kibaha | 2 | 1 | 1 | 1 | 1 | 1 | 2 |
| T0346D0 | Kibaha | 1 | 1 | 1 | 1 | 1 | 1 | 1 |
| T0348D0 | Kibaha | 1 | 1 | 1 | 0 | 1 | 1 | 1 |
| T0350D0 | Kibaha | 1 | 1 | 1 | 1 | 1 | 1 | 1 |
| T0355D0 | Kibaha | 2 | 3 | 2 | 1 | 1 | 1 | 3 |
| T0358D0 | Kibaha | 1 | 1 | 1 | 1 | 1 | 1 | 1 |
| T0360D0 | Kibaha | 2 | 3 | 2 | 2 | 1 | 1 | 3 |
| T0364D0 | Kibaha | 1 | 1 | 1 | 1 | 1 | 1 | 1 |
| T0368D0 | Kibaha | 1 | 1 | 1 | 1 | 1 | 1 | 1 |
| T0369D0 | Kibaha | 2 | 1 | 1 | 1 | 1 | 1 | 2 |
| T0402D0 | Ujiji-Kigoma | 1 | 0 | 0 | 1 | 2 | 1 | 2 |
| T0411D0 | Ujiji-Kigoma | 2 | 2 | 1 | 2 | 1 | 1 | 2 |
| T0412D0 | Ujiji-Kigoma | 1 | 1 | 0 | 1 | 1 | 1 | 1 |
| T0418D0 | Ujiji-Kigoma | 1 | 0 | 0 | 0 | 1 | 1 | 1 |
| T0421D0 | Ujiji-Kigoma | 1 | 1 | 0 | 1 | 1 | 1 | 1 |
| T0424D0 | Ujiji-Kigoma | 1 | 1 | 2 | 1 | 2 | 2 | 2 |
| T0428D0 | Ujiji-Kigoma | 1 | 0 | 0 | 1 | 1 | 1 | 1 |
| T0430D0 | Ujiji-Kigoma | 1 | 0 | 0 | 0 | 1 | 1 | 1 |
| T0436D0 | Ujiji-Kigoma | 3 | 1 | 2 | 1 | 2 | 1 | 3 |
| T0439D0 | Ujiji-Kigoma | 3 | 1 | 3 | 2 | 3 | 1 | 3 |
| T0441D0 | Ujiji-Kigoma | 1 | 1 | 0 | 1 | 1 | 1 | 1 |
| T0444D0 | Ujiji-Kigoma | 1 | 0 | 0 | 0 | 1 | 1 | 1 |
| T0447D0 | Ujiji-Kigoma | 1 | 1 | 2 | 1 | 1 | 1 | 2 |
| T0448D0 | Ujiji-Kigoma | 2 | 2 | 2 | 2 | 2 | 1 | 2 |
| T0454D0 | Ujiji-Kigoma | 2 | 0 | 0 | 0 | 1 | 2 | 2 |
| T0460D0 | Ujiji-Kigoma | 3 | 0 | 0 | 0 | 3 | 1 | 3 |
| T0466D0 | Ujiji-Kigoma | 1 | 1 | 2 | 1 | 1 | 1 | 2 |
| T0468D0 | Ujiji-Kigoma | 1 | 1 | 1 | 1 | 1 | 1 | 1 |
| T0470D0 | Ujiji-Kigoma | 1 | 0 | 0 | 0 | 2 | 1 | 2 |
| T0474D0 | Ujiji-Kigoma | 1 | 0 | 0 | 0 | 1 | 1 | 1 |
| T0475D0 | Ujiji-Kigoma | 2 | 1 | 1 | 0 | 1 | 1 | 2 |
| T0485D0 | Ujiji-Kigoma | 4 | 1 | 0 | 0 | 1 | 1 | 4 |
| T0701D0 | Mkuzi-Muheza | 1 | 1 | 1 | 1 | 1 | 1 | 1 |
| T0702D0 | Mkuzi-Muheza | 1 | 1 | 1 | 1 | 1 | 1 | 1 |
| T0704D0 | Mkuzi-Muheza | 1 | 1 | 2 | 1 | 1 | 1 | 2 |
| T0706D0 | Mkuzi-Muheza | 1 | 1 | 1 | 2 | 1 | 1 | 2 |
| T0707D0 | Mkuzi-Muheza | 1 | 2 | 2 | 0 | 2 | 1 | 2 |
| T0708D0 | Mkuzi-Muheza | 1 | 1 | 1 | 1 | 1 | 1 | 1 |
| T0709D0 | Mkuzi-Muheza | 2 | 1 | 2 | 0 | 1 | 1 | 2 |
| T0710D0 | Mkuzi-Muheza | 1 | 2 | 2 | 1 | 1 | 1 | 2 |
| T0711D0 | Mkuzi-Muheza | 3 | 0 | 0 | 0 | 3 | 1 | 3 |
| T0714D0 | Mkuzi-Muheza | 1 | 0 | 0 | 0 | 1 | 1 | 1 |
| T0717D0 | Mkuzi-Muheza | 1 | 1 | 1 | 1 | 1 | 1 | 1 |
| T0718D0 | Mkuzi-Muheza | 2 | 2 | 3 | 2 | 3 | 1 | 3 |
| T0734D0 | Mkuzi-Muheza | 1 | 1 | 2 | 1 | 2 | 1 | 2 |
| T0743D0 | Mkuzi-Muheza | 1 | 1 | 1 | 1 | 1 | 1 | 1 |
| T0747D0 | Mkuzi-Muheza | 1 | 1 | 2 | 1 | 1 | 1 | 2 |
| T0752D0 | Mkuzi-Muheza | 1 | 1 | 1 | 1 | 1 | 1 | 1 |
| T0756D0 | Mkuzi-Muheza | 1 | 1 | 1 | 1 | 1 | 1 | 1 |
| T0758D0 | Mkuzi-Muheza | 1 | 1 | 0 | 1 | 1 | 1 | 1 |
| T0761DO | Mkuzi-Muheza | 1 | 2 | 1 | 1 | 1 | 1 | 2 |
| T0767D0 | Mkuzi-Muheza | 1 | 1 | 0 | 1 | 1 | 1 | 1 |
| T0771D0 | Mkuzi-Muheza | 1 | 0 | 0 | 0 | 2 | 1 | 2 |
| T0774D0 | Mkuzi-Muheza | 3 | 1 | 2 | 1 | 3 | 2 | 3 |
| T08002D0 | Mlimba-Kilombero | 1 | 1 | 1 | 1 | 1 | 1 | 1 |
| T08008D0 | Mlimba-Kilombero | 1 | 1 | 1 | 1 | 1 | 1 | 1 |
| T08014D0 | Mlimba-Kilombero | 2 | 0 | 1 | 1 | 1 | 1 | 2 |
| T08031D0 | Mlimba-Kilombero | 1 | 1 | 1 | 2 | 2 | 1 | 2 |
| T08032D0 | Mlimba-Kilombero | 1 | 1 | 1 | 1 | 1 | 1 | 1 |
| T0803D0 | Mlimba-Kilombero | 1 | 1 | 1 | 1 | 1 | 1 | 1 |
| T0808D0 | Mlimba-Kilombero | 1 | 1 | 1 | 1 | 1 | 1 | 1 |
| T0811D0 | Mlimba-Kilombero | 1 | 2 | 2 | 1 | 1 | 1 | 2 |
| T0833D0 | Mlimba-Kilombero | 1 | 2 | 2 | 2 | 1 | 2 | 2 |
| T0834D0 | Mlimba-Kilombero | 2 | 1 | 1 | 1 | 1 | 1 | 2 |
| T0836D0 | Mlimba-Kilombero | 1 | 1 | 1 | 1 | 1 | 1 | 1 |
| T08424D0 | Mlimba-Kilombero | 1 | 1 | 1 | 1 | 1 | 1 | 1 |
| T0848D0 | Mlimba-Kilombero | 1 | 1 | 3 | 2 | 2 | 1 | 3 |
| T0852D0 | Mlimba-Kilombero | 4 | 1 | 1 | 1 | 1 | 2 | 4 |
| T0854D0 | Mlimba-Kilombero | 2 | 1 | 1 | 1 | 1 | 1 | 2 |
| T0855D0 | Mlimba-Kilombero | 1 | 1 | 1 | 1 | 1 | 1 | 1 |
| T0874D0 | Mlimba-Kilombero | 1 | 1 | 1 | 2 | 2 | 1 | 2 |
| T0880D0 | Mlimba-Kilombero | 2 | 1 | 1 | 1 | 1 | 1 | 2 |
